# The use of surfactin in inhibiting *Botrytis cinerea* and in protecting winter jujube from the gray mold

**DOI:** 10.1186/s13568-023-01543-w

**Published:** 2023-04-28

**Authors:** Peng Xiao, Xiaoyu Tian, Peng Zhu, Yangyang Xu, Chengxu Zhou

**Affiliations:** 1 School of Marine Science, Ningbo University, Ningbo, 315211 Zhejiang China; 2 College of Food and Pharmaceutical Sciences, Ningbo University, Ningbo, 315211 China; 3 College of Food and Pharmaceutical Sciences, Zhejiang-Malaysia Joint Research Laboratory for Agricultural Product Processing and Nutrition, Ningbo University, Ningbo, 315800 China

**Keywords:** Surfactin, *Botrytis cinerea*, Antifungal activity, Fatty acids, Winter jujube

## Abstract

Surfactin has the potential to be used as a food preservative. However, efficiency and action mechanism in various applications need more assessments and research. In this study, the antifungal effects and the mechanism of action of surfactin on the fungus *Botrytis cinerea* were investigated. The effects of applying surfactin for the removal of gray mold on the quality of winter jujube were investigated based on the changes in fruit fatty acids. The results showed that (1) surfactin significantly inhibited the growth of *B. cinerea*, the EC_50_ at 5 d was 46.42 mg/L. (2) Surfactin significantly reduced the disease incidence and diameter of gray mold-inoculated winter jujube in a concentration-dependent manner. For that treated with surfactin at the EC_50,_ the incidence decreased by 38.89%. (3) For *B. cinerea* under surfactin treatment, the mycelial morphology changed, the levels of total lipids and ergosterol decreased, the reactive oxygen species levels increased, and the cell integrity was completely damaged. (4) For winter jujube inoculated by *B. cinerea*, the contents of saturated fatty acids decreased and unsaturated fatty acids increased. For those under the surfactin treatments, winter jujube maintained the fatty acid composition at the level of non-inoculated groups. Mechanical injury significantly changed the fatty acid composition of winter jujube; however, surfactin not only was able to inhibit the growth of gray mold but also mitigated the adverse effects from mechanical injury. The present study demonstrated the potential applications of surfactin in the preservation of postharvest fruit quality.

## Introduction

The airborne fungus *Botrytis cinerea* is a common pathogenic fungus that causes postharvest rot of fruits and vegetables (Dean et al. 2012; An et al. 2016; Keller et al. 2003; Ren et al. 2018; Williamson et al. 2007; Qin et al. 2004). *B. cinerea* has strong adaptability and wide transmission channels (Staats et al. 2005), and It is one of the important pathogenic fungi in the fruit and vegetable industry. Rotten fruits or vegetables affected by the pathogenic fungi will produce some secondary toxic metabolites probably being harmful to human and animal health (Weiberg et al. 2013; Yang et al. 2014; Fernandez-Cruz et al. 2010; Enikova et al. 2020). Winter jujube is favored by global consumers for its rich nutrition and crisp taste (Gao et al. 2013; Wang et al. 2016; Pareek et al. 2013; Li et al. 2007). However, it is not resistant to storage. The storage period was only 2–5 days at room temperature. After harvest, winter jujube is easily infected by fungi such as *B. cinerea*, *Alternaria* spp., and *Colletotrichum gloeosporioides* (Mirzaee et al. 2014). Prevention or control of postharvest gray mold of fruits and vegetables mainly rely on chemical drugs. Although chemical fungicides can effectively inhibit the growth of or direct kill the fungi, however, on account of the residues and toxicity of chemical reagents, as well as drug resistance resulting from the continuous use of single chemical fungicides, appliying chemical fungicides on a large scale has limitations (Leroux et al. 2010; Janisiewicz et al. 2002; Jamalizadeh et al. 2011). Therefore, safer and more effective substitutes are favorable. Many previous studies showed that, metabolites from certain microorganisms can inhibit the growth of pathogens and improve the disease resistance of the plants (Dik et al. 1999; Zahavi et al. 2000; Zhang et al. 2005; Yu et al. 2008; Mora et al. 2015). Among the candidate microorganisms, species of *Bacillus* and its metabolites have attracted much attention (Akutsu et al. 1993; Hmidet et al. 2019).

Surfactin is a kind of lipopeptide biosurfactant mainly produced by *Bacillus* (Arima et al. 1968; Park et al. 2019). The bacterial cyclic lipid peptide contains seven amino acids and has an LLDLLDL chiral sequence linked by lactone bonds to β-hydroxy fatty acids of 13–15 carbon atoms (Abdelmawgoud et al. 2008; Peypoux et al. 1999). Because of the high surface activity, surfactin has been widely applied in oil recovery (Hadia et al. 2019; Yang et al. 2020), food (Gomes et al. 2012), medicine (Ahire et al. 2017; Liu et al. 2019) and agriculture (Desmyttere et al. 2019; Hazarika et al. 2019). In addition, surfaction has been reported to have good antibacterial and antifungal activities, such as inhibiting the growth of or kill *Gramella*, mold, viruses and mycoplasma (Gomaa et al. 2013; Horng et al. 2019; Płaza et al. 2013). The antibacterial activity of surfactin is thought to be closely related to its physicochemical properties (Patel et al. 2014; Buchoux et al. 2008). However, the mechanism remains unclear. In addition, it is not known whether fruit quality will be affected when applying surfactin for the control of gray mold during fruit preservation.

In this paper, the antifungal effects of surfactin on airborne fungus *B. cinerea* were studied. By observing and measuring the changes of mycelial morphology, cell membrane system and accumulation of reactive oxygen species (ROS) in *B. cinerea*, the mechanism underlying the antifungal effects was proposed. In order to evaluate the side effect on the fruit quality when surfactin was applied in inhibiting the gray mold, the changes of fatty acid compositions in winter jujube were comparatively studied. The purpose of the present study was to (1) evaluate the inhibitory effect of surfactin on *B. cinerea *in vitro* and *in vivo; (2) analyze the cell rupture mechanism involved in the process; and (3) preliminarily evaluate the validity when applying surfactin in protecting winter jujube from the gray mold.

## Materials and methods

### Biosurfactant and fruit

Surfactin was purchased from MedChemExpress LLC (Biological Company, Shanghai, China) (product number HY-129555), and the product purity was > 98%. The powdered surfactin reagent was dissolved in dimethyl sulfoxide (DMSO) to prepare the stock solution beforehand for use in the experiment.

Winter jujube was purchased from Jirong Picking Garden (Fruit Orchard, Binzhou, China). The fruit selected in the experiment were uniform in size and free from diseases, insect pests and mechanical injuries, and the brick red area of fruit skin was over 1/3 of the total area.

### Antifungal effects of surfactin on *B. cinerea *in vitro* and *in vivo

#### In vivo experiments

Freshly prepared surfactin was added to sterilized PDA to prepare media with final surfactin concentrations of 10, 20, 30, 40, and 50 mg/L. Medium supplemented with an equal volume of DMSO was used as the control. The above medium was poured into a glass petri dish, and a cake of *B. cinerea* (ACCC 36028, purchased from the Agricultural Culture Collection of China) (9 mm) was inoculated into the center of the PDA petri dish. Each experimental group was set up with three replicates. The Petri dishes were cultured at 25 °C for 5 days, and the colony diameter was measured regularly every day.

The inhibition rate was calculated as follows: *IR(%)* = *[(D*_*0*_*−D*_*t*_*)−9]/(D*_*0*_*−9)* × *100%*, where *Dt (mm)* is the colony diameter of surfactin added to the experimental group. *D*_*0*_* (mm)* is the colony diameter of the control group, and *9 (mm)* is the diameter of the *B. cinerea* cake.

Five milliliters of sterile water was added to the petri dish after 7 days of culture, and the conidia were eluted with a sterilized coating rod to prepare a spore suspension. The number of spores was adjusted to 1 × 10^5^ cfu/mL, and 2 mL of spore suspension was added to PDB medium. Freshly prepared surfactin was added to the medium to prepare experimental groups with final surfactin concentrations of 10, 20, 30, 40, and 50 mg/L, while DMSO was added to the control group. Three repetition groups were set for each concentration. Shock cultures at 120 r/min at 25 °C for 6 h were observed under an optical microscope (Olympus). Approximately 200 spores were selected to calculate the spore germination rate.

The rate of inhibition of spore germination was calculated as follows: *IR(%)* = *(C−T)/C* × *100%*, where *T(%)* is the spore germination rate of the experimental group with surfactin added, and* C (%)* is the spore germination rate of the control group.

#### In vitro experiment

The purchased fresh winter jujubes were disinfected with ethanol (75%) and air-dried for 1 h. A sterilized stainless steel rod (3 mm in diameter) was used to introduce a wound (3 mm in depth) to one side of the fruit. Each wound was inoculated with 10 µL of spore suspension (1 × 10^5^ cfu/mL), and the fruit were stored at 25 °C for 1 h. The inoculated jujubes were randomly divided into four groups. A total of 10 µL of freshly prepared surfactin at concentrations of 2 EC_50_, 4 EC_50_, and 8 EC_50_ was inoculated into the wounds. Winter jujubes without surfactin and with equal volumes of DMSO were used as the control group. Five repetitions were set for each group. Each replicate group contained 5 pieces of the fruits. The treated fruits were removed from the container and dried at room temperature. After storage at 20 °C for 5 days, the disease incidence in winter jujube and the diameters of the disease spots (mm) were recorded regularly every day (Cindi et al. 2016).

### Inhibitory mechanism of surfactin on *B. cinerea*

#### Culture and collection of mycelia and spores

One milliliter of spore suspension (1 × 10^6^ cfu/mL) was added to 100 mL of PDB medium. Shock culture was performed at 25 °C and 120 rpm for 69 h. Freshly prepared surfactin solution was added to the medium at a working concentration of 4 EC_50_, and spore suspension with an equal volume of DMSO was used as the control group. The spore suspension was incubated at 25 °C at 120 rpm for 3 h. After the culture, the mycelium was centrifuged at 4500 rpm for 5 min and washed with PBS solution (pH 7.8) three times.

Freshly prepared surfactin at a working concentration of 4 EC_50_ was added to 10 mL of PDB medium with 100 µL of spore suspension (1 × 10^6^ cfu/mL). Cells were cultured at 25 °C and 120 rpm for 3 h. After centrifugation at 5500 rpm for 5 min, spores were collected and washed with PBS solution (pH 7.8) three times.

#### Effects of surfactin on mycelial morphology

The collected mycelia were fixed with 2.5% glutaraldehyde for 24 h. After washing three times with PBS solution (pH 7.8), dehydration was carried out with anhydrous ethanol. Then, the mycelia were freeze-dried for 48 h and sprayed with gold coating for 1 min by ion sputtering. Mycelial morphology was observed by scanning electron microscopy (Hitachi, Tokyo, Japan) (Shao et al. 2015).

#### Effects of surfactin on the plasma membrane integrity of *B. cinerea*

Mycelia and spores after culture collection were stained with 10 µM propidium iodide (PI) and incubated in darkness at 37 °C for 30 min. The samples were centrifuged at 4500 rpm for 5 min and washed with PBS (pH 7.8) three times. The cells were observed under a laser scanning confocal microscope (Zeiss, Germany) (Kong et al. 2019).

#### Effects of surfactin on total lipid and ergosterol levels in mycelia

Cultured and collected mycelia were freeze-dried for 48 h. Dried mycelia (0.05 g) were ground to powder with liquid nitrogen, and 4.0 mL of a methanol: chloroform:water mixture was added (2:1:0.8). The supernatant was centrifuged after shaking for 10 min. Then, 0.2 mL of 0.9% NaCl was added. Then, 0.5 mL of 90% H_2_SO_4_ was added to the collected precipitate and heated in a water bath at 100 °C for 10 min. Then, 3 mL of vanillin phosphate solution was added, and the solution was mixed well and allowed to stand for 15 min. The absorbance value at 520 nm was determined, and the total fat content was calculated using a cholesterol standard curve (Tao et al. 2014).

Dried mycelia (0.1 g) were homogenized with liquid nitrogen, and 3 mL of 25% KOH ethanol (W/V) solution was added. After 20 min of ultrasonication, the sample was placed in a water bath at 80 °C for 2 h. One milliliter of sterile water and 3 mL of n-heptane were mixed for 5 min and then allowed to stand and stratify. The absorbance of the upper organic phase was measured at 281 nm and 230 nm.

The formula for calculating the ergosterol content was as follows: *C(%)* = *[(A*_*281/290*_*)−(A*_*230/518*_*)]/W*, A_281_ and A_230_ were the absorbance of ergosterol and 24 (28) dehydroergosterol, respectively, and W is the net dry weight (g) of the thallus (Johnston et al. 1984).

#### Changes in the ROS levels of *B. cinerea* under surfactin stress

Mycelia and spores were cultured and collected for later use. ROS detection kits (Solarbio Biology Engineering Institute, Beijing, China) were used to determine the ROS levels of mycelia and spores treated with surfactin in accordance with the operating manual.

### Changes in the fatty acid composition of winter jujube as affected by gray mold and surfactin treatment

The winter jujube was disinfected with ethanol (75%) and air-dried for 1 h. A sterilized stainless steel rod (diameter: 3 mm) was used to introduce a wound (depth: 3 mm) on one side of the fruit. These punched fruits were divided into four groups. Three groups were inoculated with 10 µL of spore suspension (1 × 10^5^ cfu/mL). The inoculated fruit were stored at 25 °C for 1 h. Then, two groups of inoculated fruits were injected with 10 µL of surfactin solutions with working concentrations of 2 EC_50_ and 8 EC_50_ into the wound surface. Another contaminate group was treated with the same volume of DMSO and was set as the inoculated control group. The wounded fruit that were not treated with spore suspension or surfactin were defined as the mechanical injury group. Five replicates of each group were set, and five fruits in each replicate contained five fruits. After drying at room temperature, all the groups were stored at 20 °C for 5 days.

Fatty acid analysis was conducted according to the method of Metcalfe (Metcalfe et al. 1996). To sample the fruit tissue for fatty acid analysis, a fruit digger was used to extract pulp from around the wound. The same amount of pulp was sampled from the fruit part that was not punched or inoculated. These samples were grouped into nonmechanical injury groups. 2 g of the samples from each group was freeze dried. 100 mL of the frozen dried sample was ground to powder, and 2 mL of chloroform:methanol solution (V/V = 1:1) was added. After ultrasonic crushing for 10 min, centrifugation was performed at 3000 r min^−1^ for 10 min. The supernatant was collected and dried in a rotary evaporator (Great Wall Technology & Trade Co., LTD., Zhengzhou, China), and this process was repeated twice. Then, 100 μL of 1 μg μL^−1^ C19:0 fatty acid internal standard and 2 mL of 5–6% potassium hydroxide methanol solution (V/V = 4:1) were added, and saponification was performed in a water bath at 60 °C for 2 h. After cooling, 6 mL of chloroform n-hexane mixture (V/V = 1:4) was added for extraction, and the mixture was centrifuged at 3000 r min^−1^ for 10 min; the supernatant was collected and evaporated in a rotary evaporator. Then, 0.5 mL of boron trifluoride methanol solution was added to the water bath at 60 °C for 1 h. After cooling, 6 mL of analytical grade n-hexane was used for extraction. After collecting the supernatant, 3 g of anhydrous sodium sulfate was added, and the solution was allowed to stand for 3 h. The supernatant was cyclically steamed again and analyzed by GC–MS (NYSE: A, Palo Alto, America) with 1 mL of chromatographic n-hexane at constant volume.

Based on the mass spectrometry analysis of each component, the molecular weight was determined. The mass spectra of the ion fragments were combined and searched against the NIST library and WILEY library. The percentage of fatty acids was calculated by the area normalization method. Based on the initial concentration and peak area of the internal standard C19:0 fatty acid and the peak area of each component to be measured, the absolute content of fatty acid components was calculated, and the response factor was calculated as 1.

### Data analysis

Statistical analysis was carried out using the social science statistical software package (SPSS). The EC_50_ was obtained by linear regression between the surfactin concentration and rate of inhibition of *B. cinerea* using SPSS. One-way ANOVA (LSD) was used to determine the differences between the surfactin treatment group and the control group. Principal component analysis (PCA) was used to analyze the differences in fruit fatty acids among the different treatment groups. The significance level was set as *P* < 0.05. Origin software was used for plotting the data.

## Results

### In vitro* and *in vivo experiments

#### Antifungal effects of surfactin on *B. cinerea *in vitro

**Fig. 1:**
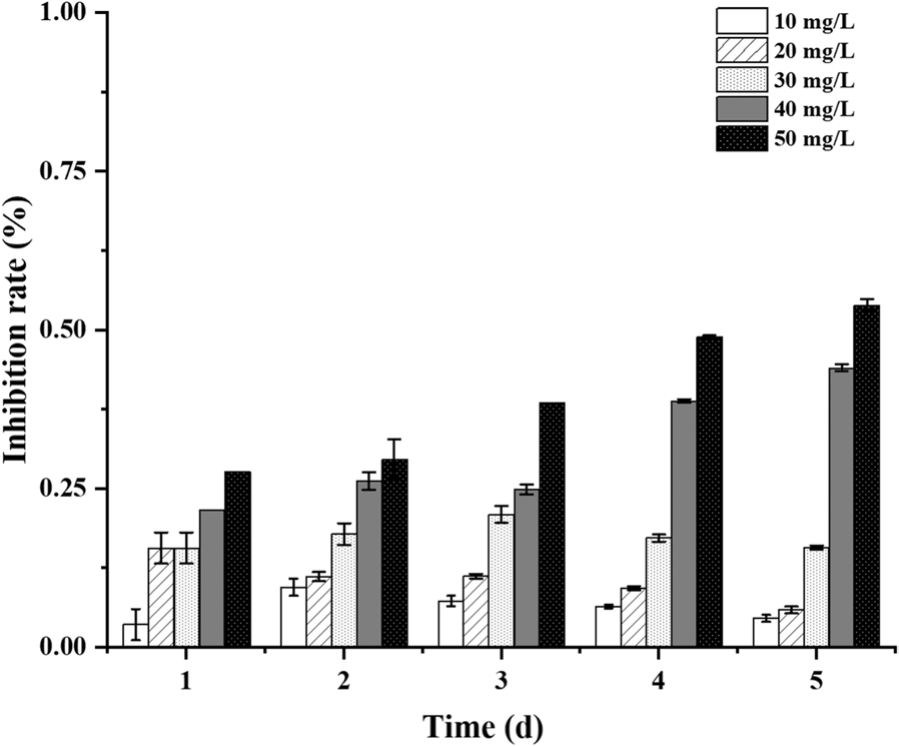
Inhibitory effects of different concentrations of surfactin on mycelial growth of *B. cinerea*. The error bars represent the SEs of the three replicates

The rates of inhibition of mycelial growth of *B. cinerea* under surfactin stress are shown in Fig. 1 The results showed that the inhibition was concentration dependent over 5 days. The surfactin treatment groups (10 mg/L, 20 mg/L, 30 mg/L) showed the highest inhibition rates at 2 d, 1 d and 3 d, with 9.6%, 15.66% and 20.98% inhibition, respectively; at the end of the experiment (5 d), the inhibition rates decreased to 4.61%, 5.92% and 15.79%, respectively. The inhibition rates in the surfactin treatment groups (40 mg/L, 50 mg/L) increased significantly within 5 days. At 5 d, the inhibition rates in the 40 mg/L and 50 mg/L surfactin treatment groups were 44.08% and 53.95%, respectively, showing a significant concentration dependence (*P* < 0.05). The EC_50_ was 46.42 mg/L for 5 d.

**Fig. 2:**
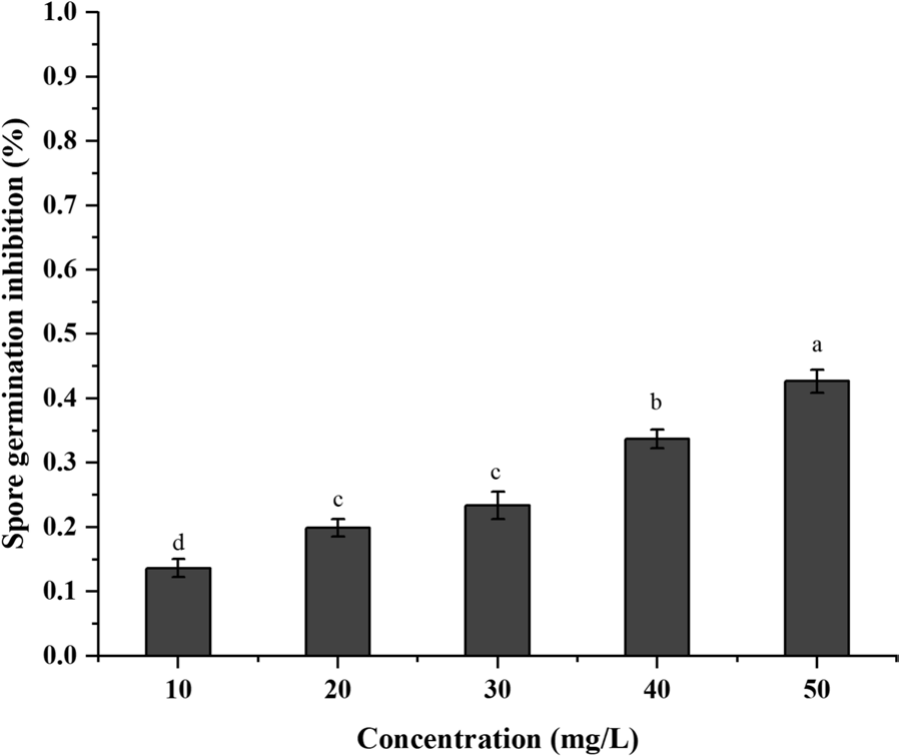
Inhibition effects of different concentrations of surfactin on spore germination of *B. cinerea*. The error bars represent the SEs of the three replicates

Surfactin significantly reduced the spore germination rate of *B. cinerea* (Fig. 2). The inhibition rate was dependent on the surfactin concentration. The rate of inhibition in the 10 mg/L surfactin treatment group was low, only 13.63%. There was no significant difference between the 20 mg/L and 30 mg/L surfactin groups (P > 0.05). When the concentration of surfactin increased to 40 mg/L, the rate of inhibition increased to 33.71%, which was 2.47 times that of the 10 mg/L surfactin treatment group. The rate of inhibition in the 50 mg/L surfactin treatment group was 42.67%, which was 3.13 times that in the 10 mg/L group. The surfactin treatment groups (40 mg/L, 50 mg/L) showed a significant concentration dependence for the rate of inhibition (*P* < 0.05).

#### Inhibitory effects of surfactin on *B. cinerea* in vivo

**Fig. 3:**
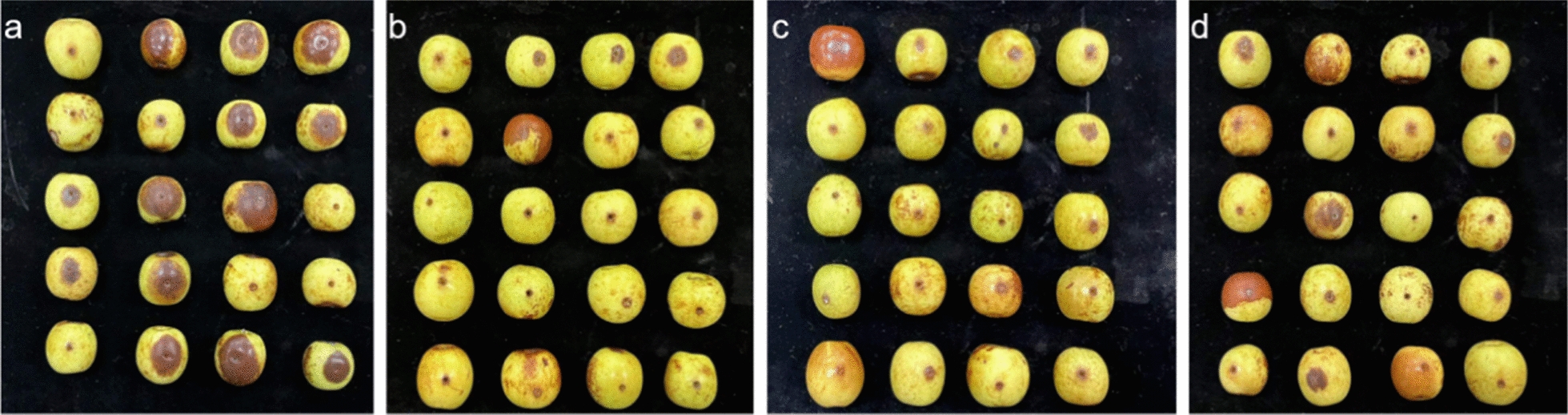
Disease characteristics of winter jujube inoculated by gray mold treated with surfactin at different concentrations for 3 days **a**: control group without surfactin treatment; **b** EC_50_ surfactin treatment group; **c**: 4 EC_50_ surfactin treatment groups; **d**: 8 EC_50_ surfactin treatment groups

**Fig. 4:**
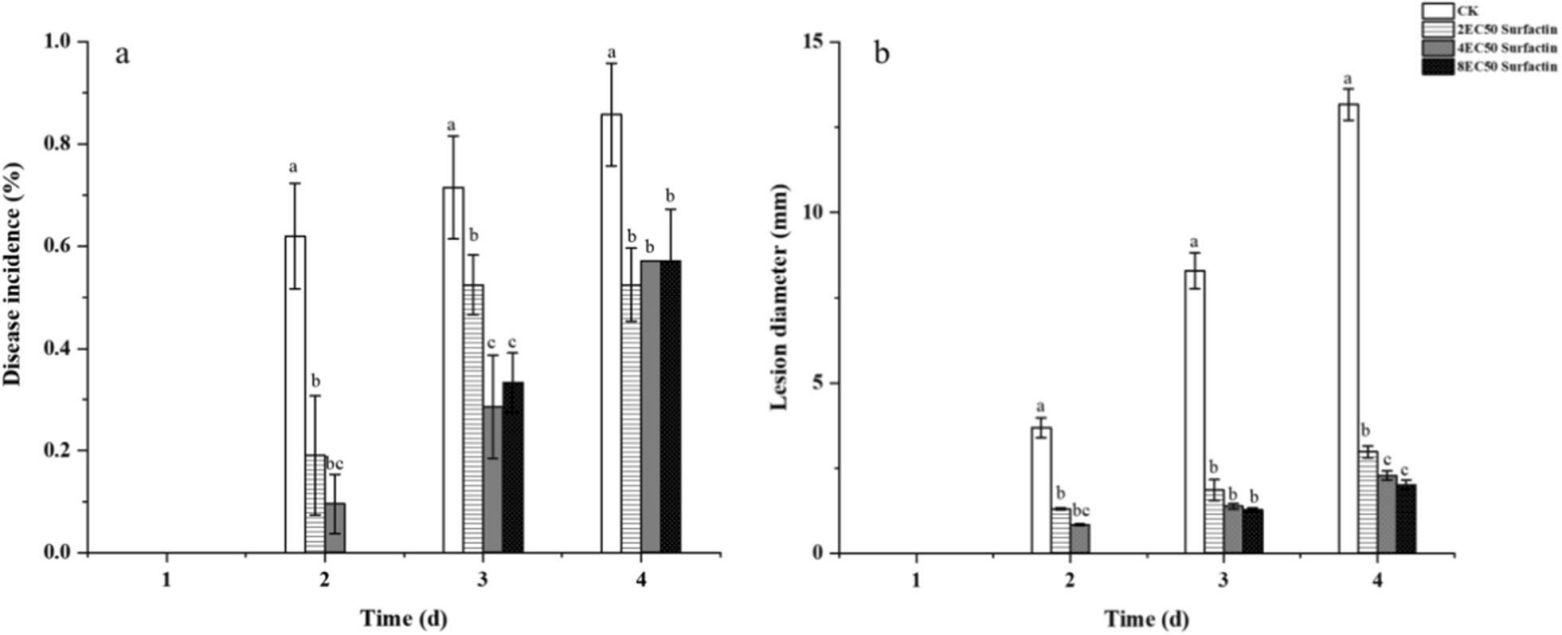
Disease incidences of winter jujube inoculated by gray mold treated with surfactin at different concentrations: **a** percentage of disease incidence and **b** lesion diameter of gray mold patches on winter jujube. The error bars represent the SEs of the three replicates

As shown in Fig. 3, surfactin reduced the fruit disease incidence and spot diameter with artificial *B. cinerea* inoculation. The incidence in the surfactin treatment group was significantly lower than that in the control group (*P* < 0.05, Fig. 4a). At 2 d, the disease incidence was 61.9% in the control group and 19.05% and 9.52% in the 2 EC_50_ and 4 EC_50_ surfactin treatment groups, respectively. The disease incidence in the 8 EC_50_ surfactin treatment group was 0%. After 3 days of storage, the disease incidence in the 8 EC_50_ surfactin treatment group increased to 33.33%, and there was no significant difference between the 8 EC_50_ and the 4 EC_50_ surfactin treatment group (P > 0.05). At 4 d, the disease incidence rate with surfactin treatment at 2 EC_50_ was the lowest (52.38%). The disease incidence was 57.14% in the 4 EC_50_ and 8 EC_50_ surfactin treatment groups, showing no significant difference.

At 2 d, the lesion diameters in the 2 EC_50_, 4 EC_50_ and 8 EC_50_ surfactin treatment groups were 3.69, 1.31 and 0.83 mm, respectively, showing a significant difference (*P* < 0.05, Fig. 4b). At 3 d, the lesion diameters in the 2 EC_50_, 4 EC_50_ and 8 EC_50_ surfactin treatment groups were 1.86, 1.38 and 1.29 mm, respectively, which were 22.41%, 11.67% and 15.52% of those in the control group, respectively. At 4 d, the surface diameters in the 2 EC_50_, 4 EC_50_ and 8 EC_50_ treatment groups were 22.60%, 17.36% and 15.37% of that in the control group, while the surface diameters in the three surfactin treatment groups were 2.98, 2.29 and 2.02 mm, respectively, showing a significant difference compared with the control group (*P* < 0.05). The surfactin concentration was positively correlated with the diameter of the disease spots.

### Antifungal mechanisms of surfactin

#### Effects of surfactin on the mycelial morphology of* B. cinerea*

**Fig. 5:**
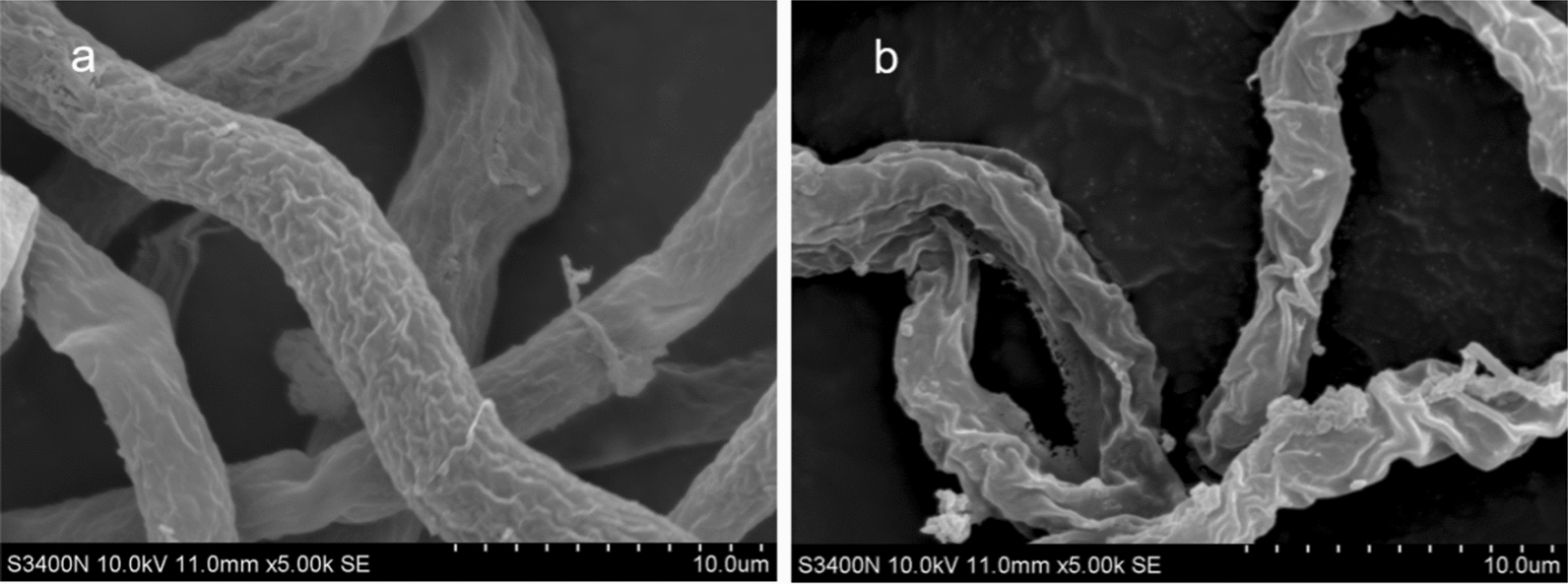
The mycelial morphology of *B. cinerea* in the control group (**a**) and in the 4 EC_50_ surfactin treatment groups (**b**)

Through scanning electron microscopy, we observed that the mycelia in the control group were complete, smooth and normal without fracture (Fig. 5a, red arrow). The mycelia in the 4 EC_50_ surfactin treatment group were completely distorted and wrinkled (Fig. 5b, red arrow), with no intact mycelium observed.

#### Effects of surfactin on the membrane integrity of* B. cinerea*

**Fig. 6:**
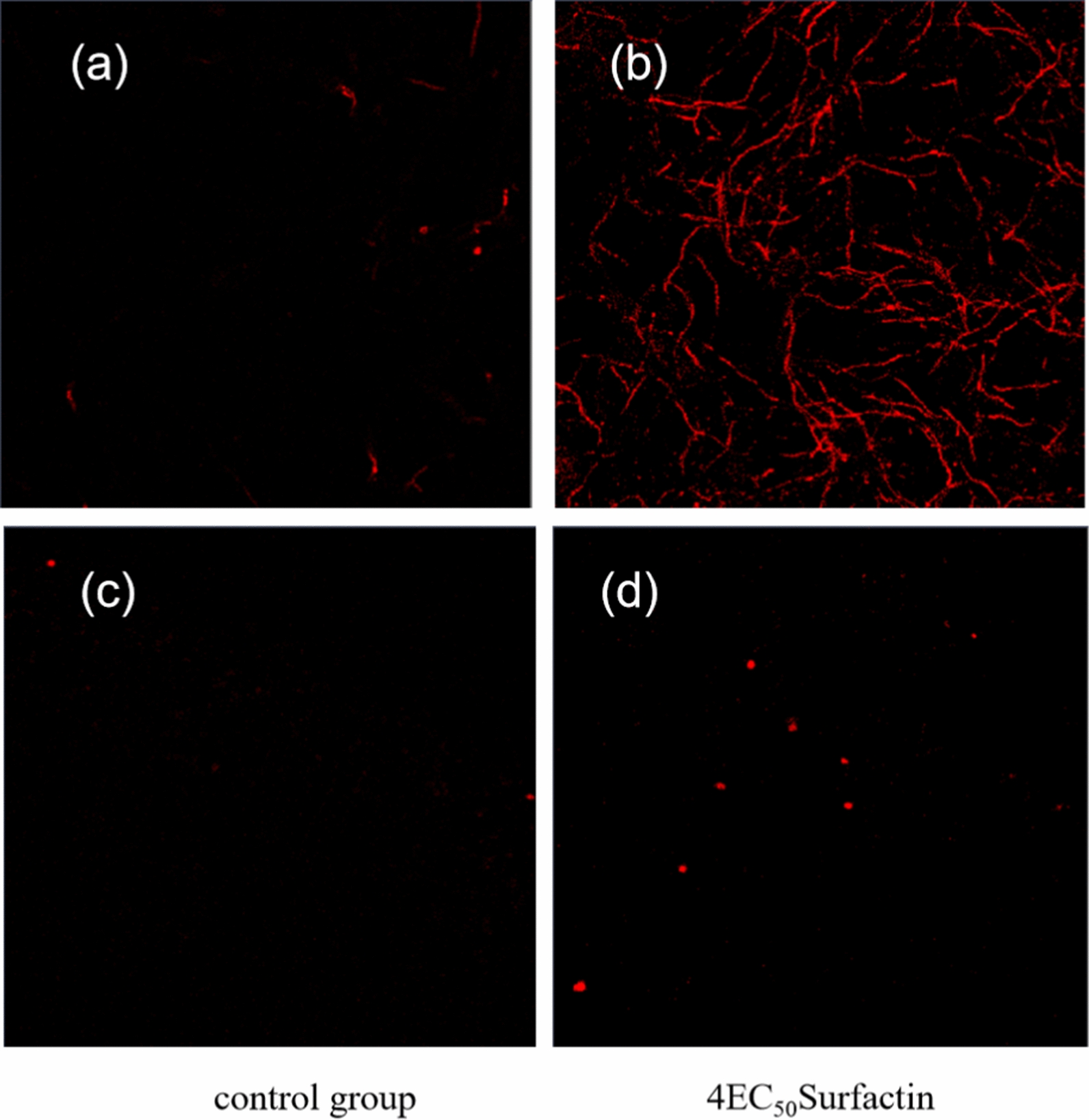
Different levels of propidium iodide staining of mycelia (**a**, **b**) and spores (**c**, **d**) of *B. cinerea* in the control and 4 EC_50_ surfactin groups. Images were obtained by laser scanning confocal microscopy at 10 × magnification

PI is a common nucleic acid dye that can penetrate the membrane of dead cells and stain the nucleus to appear red in a fluorescent environment. The effects of different concentrations of surfactin on the cell integrity of *B. cinerea* are shown in Fig. 6. There was a small amount of red fluorescence in the control group (Fig. 6a), while the red fluorescence in the surfactin-treated group covered almost the entire visual field (Fig. 6b). Among the spores, only a very small number were stained by PI in the control group (Fig. 6c), while the number of spores stained by PI in the surfactin-treated group was significantly higher than that in the control group (Fig. 6d).

#### Effects of surfactin on the total lipid and ergosterol levels of *B. cinerea*

**Fig. 7:**
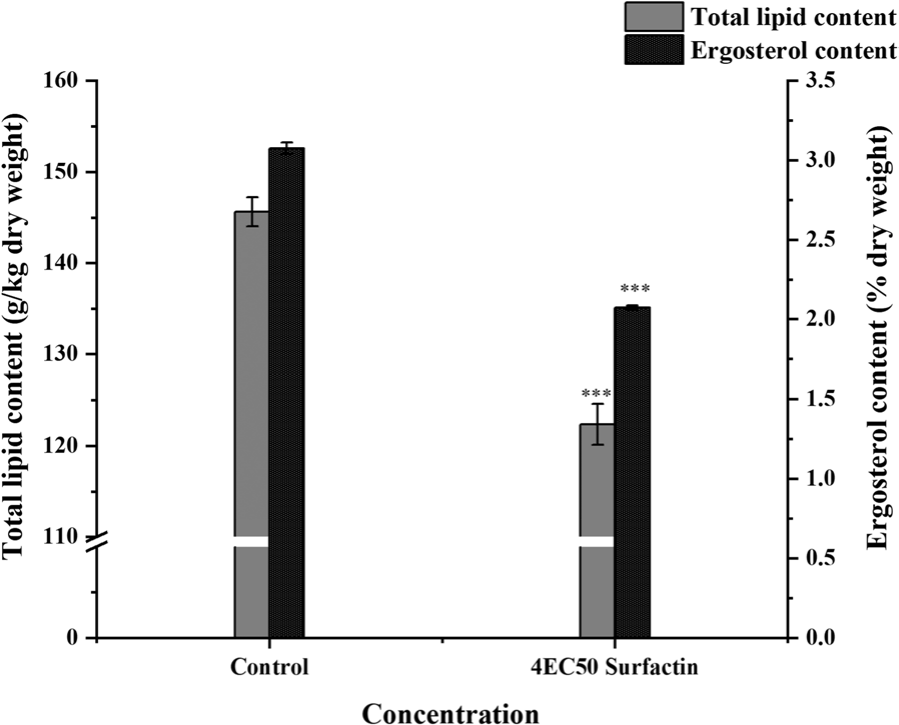
Total lipid and ergosterol levels in mycelia of *B. cinerea* in the control and 4 EC_50_ surfactin groups. The error bars represent the SEs of 3 replicates

Surfactin significantly reduced the total lipid and ergosterol levels of *B. cinerea* (Fig. 7). The total fat content in the control group was 145.62 g/kg and that in the 4 EC_50_ surfactin treatment groups was 122.36 g/kg, showing a significant difference (*P* < 0.05). The ergosterol content in the control group was 3.07%, and that in the 4 EC_50_ surfactin treatment groups was decreased to 2.07%, showing a significant difference (*P* < 0.05).

#### Effects of surfactin on the ROS content of *B. cinerea*

**Fig. 8:**
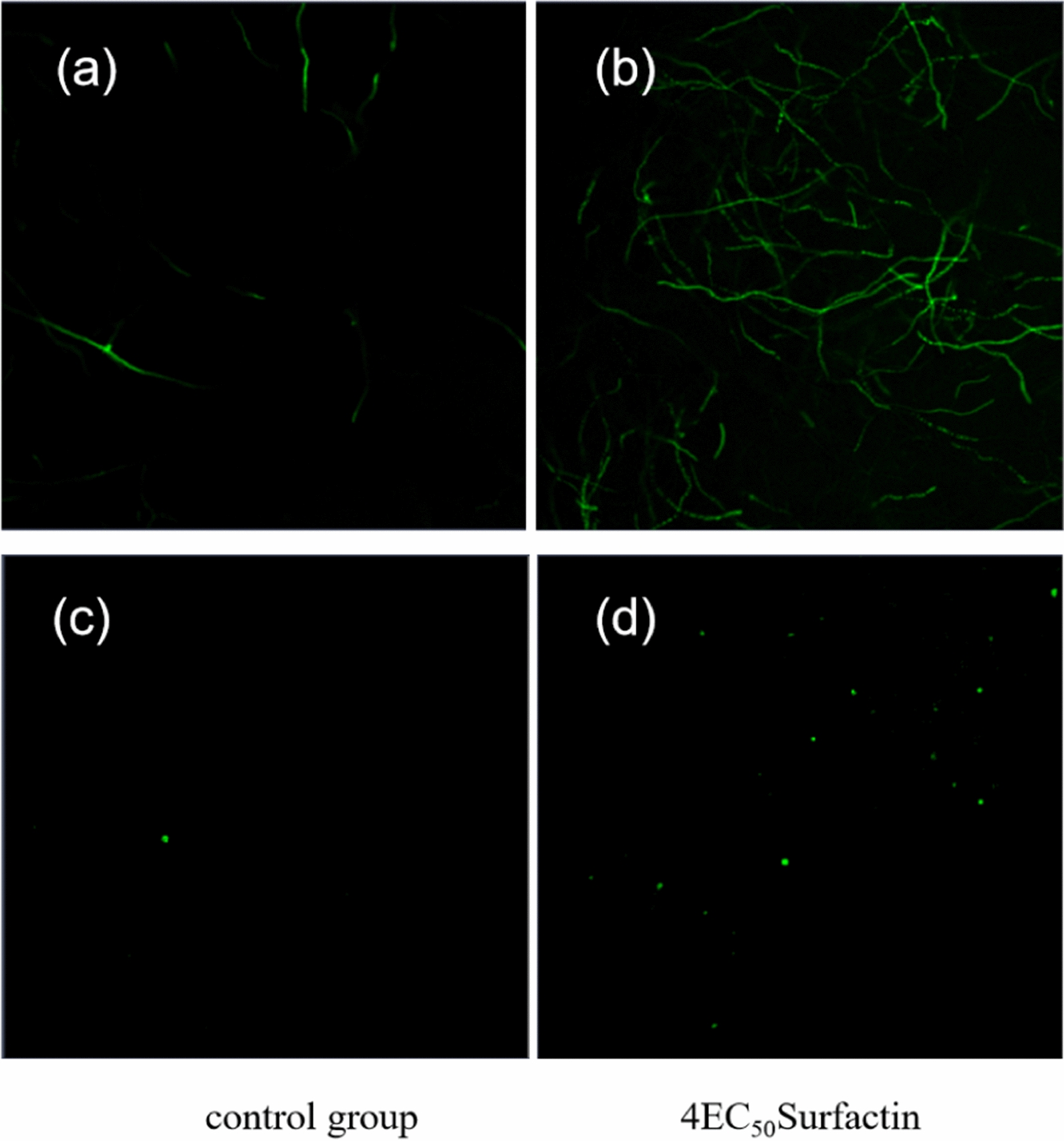
Different ROS contents of mycelia (**a**, **b**) and spores (**c**, **d**) of *B. cinerea* in the control and 4 EC_50_ surfactin groups. Images were obtained by laser scanning confocal microscopy at 10 × magnification

The fluorescent probe DCFH-DA can be hydrolyzed to DCFH by esterase across the cell membrane, which can be oxidized by intracellular ROS to generate DCF with green fluorescence. The effects of surfactin on the ROS content of *B. cinerea* are shown in Fig. 8. In mycelia, there was a small amount of intermittent green fluorescence in the visual field of the control group (Fig. 8a), while in the 4 EC_50_ surfactin-treated group, there was a large amount of green fluorescence with strong continuity (Fig. 8b). In spores, the amount of green fluorescence in the 4 EC_50_ surfactin treatment groups was significantly higher than that in the control group (Fig. 8c, d).

### Changes in fatty acid composition in winter jujube as affected by gray mold and surfactin treatment

In total, 12 fatty acids were identified in different winter jujube groups, including 5 saturated fatty acids (SFAs), 5 monounsaturated fatty acids (MUFAs) and 2 polyunsaturated fatty acids (PUFAs) (Table 1). SFAs accounted for the highest proportion (48.31–54.87%) in each group. The dominant SFAs were palmitic acid (C16:0) and stearic acid (18:0), accounting for 32.00–36.06% and 14.66–17.37%, respectively. The SFA content in the mechanical injury group was the highest, and those of C16:0 and C18:0 were 35.87% and 17.37%, respectively. The SFA content in the inoculated control group was the lowest, and those of C16:0 and C18:0 were 32.00% and 14.66%, respectively. Compared with the nonmechanical injury group, the increase in C18:0 in the mechanical injury group was statistically significant (*P* < 0.05). Compared with the mechanical injury group, the SFA content in the 2EC_50_ group was statistically similar (*P* > 0.05), while in the 8EC_50_ group, it decreased significantly (*P* < 0.05).

**Table 1:** Fatty acid composition (%) of winter jujube in different treatment groups

Fatty acids	Nonmechanical injury group (A)	Mechanical injury group (B)	Inoculated control group (C)	2EC_50_ Surfactin (D)	8EC_50_ Surfactin (E)
C14:0	1.39 ± 0.28^a^	1.47 ± 0.14^a^	1.05 ± 0.11^a^	1.20 ± 0.24^a^	1.28 ± 0.25^a^
C14:1(n-3)	0.45 ± 0.02^b^	0.77 ± 0.01^a^	0.43 ± 0.00^b^	0.26 ± 0.16^b^	0.41 ± 0.01^b^
C16:0	32.59 ± 0.23^ab^	35.87 ± 0.23^ab^	32.00 ± 0.46^b^	36.06 ± 1.92^a^	32.37 ± 0.58^ab^
C16:1(n-7)	12.57 ± 0.18^c^	14.8 ± 0.08^a^	14.63 ± 0.55^a^	14.10 ± 0.06^ab^	13.28 ± 0.28^bc^
C17:0	0.53 ± 0.01^a^	0.16 ± 0.20^ab^	0.13 ± 0.16^ab^	–^b^	0.55 ± 0.03^a^
C18:0	15.23 ± 0.34^bc^	17.37 ± 0.19^a^	14.66 ± 0.25^c^	15.91 ± 0.45^b^	14.78 ± 0.45^c^
C18:1(n-9)	7.50 ± 0.09^c^	7.12 ± 0.07^c^	10.92 ± 0.48^a^	9.04 ± 0.21^b^	8.86 ± 0.24^b^
C18:1(n-7)	8.54 ± 0.12^b^	7.82 ± 0.07^c^	7.04 ± 0.05^d^	8.40 ± 0.19^bc^	9.40 ± 0.26^a^
C18:2(n-6)	12.18 ± 0.09^b^	8.74 ± 0.14^d^	13.49 ± 0.28^a^	11.74 ± 0.13^bc^	11.49 ± 0.04^c^
C18:3(n-3)	6.06 ± 0.20^a^	4.50 ± 0.25^ab^	4.35 ± 0.13^ab^	2.86 ± 1.75^b^	4.99 ± 0.26^ab^
C22:0	1.75 ± 0.03^a^	–^c^	0.47 ± 0.58^bc^	–^c^	1.13 ± 0.70^ab^
C22:1(n-9)	1.21 ± 0.19^ab^	1.37 ± 0.08^a^	0.83 ± 0.53^ab^	0.41 ± 0.50^b^	1.47 ± 0.12^a^
SFA	51.49 ± 0.28^bc^	54.87 ± 0.27^a^	48.31 ± 0.32^d^	53.18 ± 2.13^ab^	50.11 ± 0.45^cd^
MUFA	30.27 ± 0.56^c^	31.89 ± 0.03^b^	33.85 ± 0.5^a^	32.21 ± 0.82^b^	33.42 ± 0.65^ab^
PUFA	18.24 ± 0.29^a^	13.24 ± 0.29^c^	17.85 ± 0.18^a^	14.61 ± 1.7^bc^	16.47 ± 0.3^ab^
UFA	48.51 ± 0.85^bc^	45.13 ± 0.31^c^	51.69 ± 0.69^a^	46.82 ± 2.52^cd^	48.89 ± 0.95^ab^

Lowercase letters show the statistical significance of the difference between groups

Overall, MUFAs were significantly higher than PUFAs (*P* < 0.05). These two groups of unsaturated fatty acids (UFAs) accounted for 30.27–33.85% and 13.24–18.24%, respectively (Table 1). The dominant UFAs were palmitoleic acid (C16:1), oleic acid (C18:1), linoleic acid (C18:2) and α-linolenic acid (C18:3). UFAs in the mechanical injury group were the lowest, among which the percentages of C16:1, C18:1, C18:2 and C18:3 were 14.8%, 14.94%, 8.74% and 4.50%, respectively. UFAs in the inoculated control group were the highest, among which the percentages of C16:1, C18:1, C18:2 and C18:3 were 14.63%, 17.96%, 13.49% and 4.35%, respectively. Compared with the mechanical injury group, UFAs in the 2EC_50_ group were statistically the same (*P* > 0.05), while those in the 8EC_50_ group were significantly higher (*P* < 0.05) and were similar to those in the nonmechanical injury group (*P* < 0.05).

**Fig. 9:**
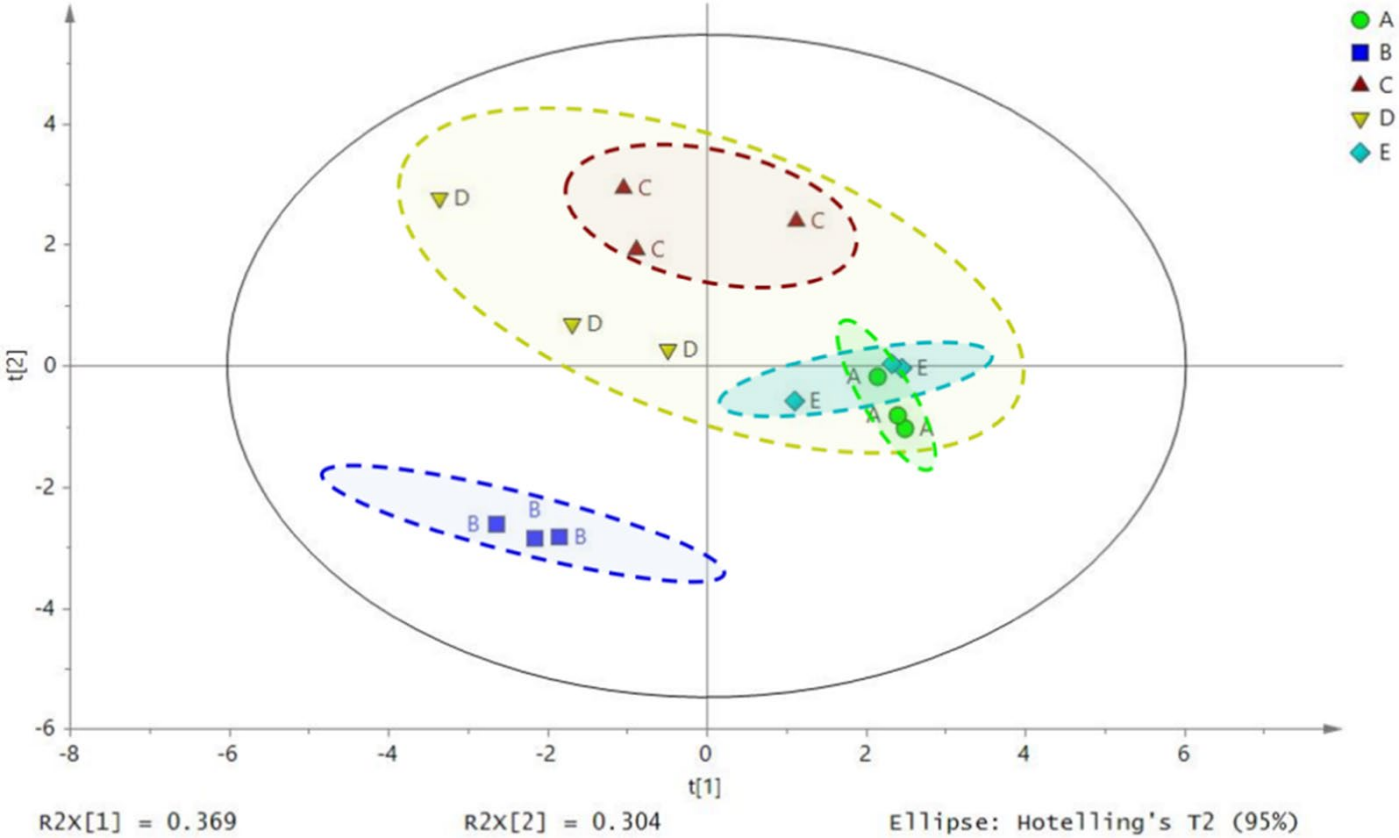
Principal component analysis of the fatty acid content of winter jujube in different groups. **A** nonmechanical injury group; **B** mechanical injury group; **C** gray mold-inoculated group; **D** 2EC_50_ surfactin; **E** 8EC_50_ surfactin

Principal component analysis (PCA) was conducted on the composition of fatty acids in different groups (Fig. 9). The contribution rate of the first component was 36.9%, that of the second component was 30.4%, and the total contribution rate of the two components was 67.3% (Fig. 9). The mechanical damage group (B) was further different from the other groups. The inoculated control group (C) and the two surfactin treatment groups (D, E) were discrete. 8 EC_50_ surfactin treatment group (E) and the nonmechanical injury group (A) partially overlapped, showing no significant difference.

## Discussion

In the present study, surfactin had a significant inhibitory effect on *Botrytis cinerea*. The higher the concentration was, the stronger the inhibition. *Bacillus* species produce a variety of antibacterial compounds, among which cyclolipopeptides (LPs) are a class of antibacterial substances with surfactant properties, mainly include iturin, fengycin and surfactin. LPs have bacteriostasis, growth promotion and defense-related enzyme activity (Vanittanakom et al. 1986; Ongena et al. 2008; Liu et al. 2010; Zhang et al. 2010; Goussous et al. 2017). Płaza et al. (2013) isolated surfactin from *Bacillus subtilis* cultured with molasses and explored its antibacterial activity in vitro. The results showed that, it had a mild inhibitory effect on several common plant pathogens, such as *B. cinerea* A258, *Nuclear discomycetes* K2291 and *Colletotrichum gloeosporioides* A259.

Cell membrane is the most important target of active bacteriostatic substances of *Bacillus* (Zouari et al. 2016; Senna et al. 2017; Zhang et al. 2013; Guo et al. 2014). In the present study, surfactin caused deformation of the mycelia of *B. cinerea*, showing the cell permeability increased and resulting the leakage of small molecules or ions from the cells. Jin et al. (2018) found that bacillomycin D, a secondary metabolite of *Bacillus velezensis* HN-2, caused irreversible damage to the cell membrane of hyphae and spores of anthrax, leading exudations of cell contents. Wang et al. (2020) found that surfactin and fengycin B obtained from *Bacillus pumilus* W-7 could inhibit *Phytophthora filaria* by inducing mycelial deformation. Xin et al. (2019) isolated the alkaloid antofine from *Cynanchum atratum* and found that it significantly damaged the membrane integrity of *Penicillium digitatum*. In this study, the fluorescence intensity of the PI-stained cells was significantly increased in the surfactin-treated groups, indicating that surfactin damaged the integrity of the cell membrane of *B. cinerea* (Setiawati et al. 2017; Zhang et al. 2019).

Lipids are the main component of the cell membrane. Reduction in lipid content usually hinders the transport of lipid soluble substances, resulting in the loss of selective permeability of the cell membrane (Wei et al. 2010). Helal et al. (2006) found that citronella significantly reduced the total lipid content of *Aspergillus niger*, leading to dysfunction of cell membranes. Ergosterol is a unique component of the fungal cell membrane and plays important roles in protecting cell membrane integrity, fluidity and material transport (Verma et al. 2002; Li et al. 2012). Siahmoshteh et al. (2018) showed that the culture filtrate of *Bacillus subtilis* and *Bacillus amyloliquefaciens* affected ergosterol synthesis of parasitic *Aspergillus* NRRL2999, resulting in impaired cell membrane integrity. In this study, we found that surfactin significantly reduced the total lipid and ergosterol contents of *B. cinerea*, showing that cell membranes are important targets for surfactin.

ROS play an important role in cell viability. Excessive ROS production can cause oxidative damage to compounds in cells, inhibit enzymes, destroy cell membranes, and lead to cell dysfunction or death (Pi et al. 2010; Shi et al. 2012; Marchi et al. 2011). Massawe et al. (2018) showed that bacteriostatic substances produced by *Bacillus velezensis* VM11 induced the accumulation of ROS in the mycelial cells of *Sclerotinia sclerotiorum*, leading to the cell damage. In this study, we found that surfactin can cause high accumulation of ROS in *B. cinerea* cells, suggesting that lipids, DNA and proteins in mycelia and spore cells may have undergone severe oxidative damage.

Changes in fatty acids are related to cell membrane stability, fluidity and structure. The variations are are closely correlated with the quality of fruit and vegetable (Meyer et al. 2010; Zhang et al. 2018). Chen et al. (2011) found that the lipoxygenase (LOX) activity increased in longan peel after the infection by *Phomopsis longanae* Chi. In the meanwhile, the relative content of UFA decreased and that of SFA increased. From the results of our study, the compositions of fatty acids were significantly changed by different treatments. Mechanical injury resulted in varied compositions of fatty acids that were significantly different from the other groups, including nonmechanical injury groups and the gray mold-inoculated groups (both the control and the surfactin-treated groups). However, surfactin treatment, especially with the higher concentration (8EC_50_), helped the fruit maintain the fatty acid composition similar to that of the nonmechanical injury groups. The surfactin treatment group was mechanically injured before being inoculated with mold and surfactin. This phenomenon demonstrated that surfactin not only inhibited the growth of gray mold but also maintained the fatty acid quality of the fruit when it was mechanically injured. The fatty acid composition could be changed by physical treatment, such as a lower temperature. Cao et al. (2009) showed that methyl jasmonate can reduce the degree of chilling injury in loquat by reducing the fatty acid composition of the cell membrane. Gray mold contamination significantly decreased the composition of SFA, while in the surfactin treatment groups, SFA contents increased back to the level of the non-inoculated groups. The contents of UFAs, such as C18:1 and C18:2, were also significantly affected by surfactin treatments, showing that lipid metabolism related to these fatty acids in the interaction of gray mold and mechanically injured fruit was altered by surfactin. These results suggest that the metabolic target for further studies in the mechanism of gray mold contamination.

## Data Availability

All data generated and analyzed during this study are included in this published article.
